# Effect of Dietary Insect Meal and Grape Marc Inclusion on Flavor Volatile Compounds and Shell Color of Juvenile Abalone *Haliotis iris*

**DOI:** 10.1155/2023/6628232

**Published:** 2023-07-18

**Authors:** Natalia Bullon, Andrea C. Alfaro, Nazimah Hamid, Sara Masoomi Dezfooli, Ali Seyfoddin

**Affiliations:** ^1^Drug Delivery Research Group, School of Science, Auckland University of Technology, Auckland, New Zealand; ^2^Aquaculture Biotechnology Research Group, School of Science, Auckland University of Technology, Auckland, New Zealand; ^3^Department of Food Science, Auckland University of Technology, Auckland, New Zealand

## Abstract

Almost 60% of the fish meal produced globally is used in aquaculture feeds. Fish meal production relies on finite wild-marine resources and is considered as an unsustainable ingredient. Insect meal (IM) is considered a sustainable source with high levels of protein suitable for growth promotion. Grape marc (GM) is a waste byproduct of the winery industry rich in pigments with antioxidant capacity. However, the inclusion of both ingredients can affect the flavor of the meat of abalone and the color of the shell due to different nutritional profiles. The aim of this study was to evaluate the effect of the dietary inclusion of IM and GM on the flavor volatile compounds and shell color of the juvenile *Haliotis iris* in a 165-days feeding trial. Abalone were offered four experimental diets with different levels of IM and GM inclusion and a commercial diet (no IM or GM). Soft bodies of abalone were used to characterize volatile compounds using solid-phase microextraction gas chromatography–mass spectrometry, and color changes were analyzed in ground powder of abalone shells using color spectrophotometry 400–700 nm (visible). The results showed 18 volatile compounds significantly different among the dietary treatments. The inclusion of IM did not significantly affect the flavor volatile compounds detected, whereas the inclusion of GM reduced volatile compounds associated with lipid-peroxidation in abalone meat. The inclusion of IM and GM did not significantly affect the lightness nor the yellowness, blueness, redness, and greenness of the ground shells. The supplementation of abalone feeds with GM can help to reduce off-flavour compounds which may extend shelf-life of raw abalone meat.

## 1. Introduction

In recent years, there has been a growing interest to replace the fish meal in aquafeeds across the aquaculture industry globally. The reasons behind this quest are the increasing fish meal price (300% in the last 30 years) [1] and the use of wild marine fish stocks to produce fish meal which is deemed unsustainable. Alternative ingredients have been widely investigated to replace fish meal in aquaculture feeds [2]. One example of an alternative ingredient is insect meal (IM), which is seen as a good source of protein and lipids [2]. Insects have fast growth, reproduce easily, convert low-grade organic matter into high-value protein and fat, and do not require cultivable land [3]. These characteristics make IM a favorable option, which is regarded as more sustainable than fish meal [4, 5]. Different insect species have been included in aquafeeds. Yellow mealworm (*Tenebrio molitor*) is one of the most studied species with great potential for industrialization and high digestibility indexes [6]. *T. molitor* has been included in diets for Nile tilapia (*Oreochromis niloticus*) [6], European sea bass (*Dicentrarchus labrax*) [7], rainbow trout (*Oncorhynchus mykiss*) [8], pacific white shrimp (*Litopenaeus vannamei*) [9], and mandarin fish (*Siniperca scherzeri*) [10]. Aquafeeds including IM for abalone have included silkworm pupae meal [11] promoting superior weight and shell gain compared to the fish meal-based diets.

Another ingredient which has potential for aquafeeds is grape marc (GM). GM is an agro-industrial byproduct which consists of grape skins, stalks, and seeds from the winemaking process of *Vitis vinifera*. Almost 18%–20% of all harvested grapes used for wine production end up as GM [12]. Due to the large amount of waste produced annually, GM disposal has become problematic [13]. GM has been used as feed for ruminants [14–16] due to the high-dietary fiber content and elevated levels of bioactive compounds, such as tocopherol and polyphenols [17]. GM has high levels of polyunsaturated fatty acids (PUFAs), mostly high levels omega-6 fatty acids, such as linoleic acid (C18:2n−6), oleic acid (C18:1n−9) [18], and saturated fatty acids (SFA), such as stearic acid (C18:0) and palmitic acid (C16:0) [19].

Changes in animal diet affect the physical [20] and chemical characteristics of the meat [21]. These characteristics play an important part in consumer perception of quality and therefore influence purchase price [22, 23]. From these characteristics, the flavor profile, which is mostly shaped by the volatile compounds, is the key difference for more competitive market advantages [24], representing a signature for high-quality products. Volatile compounds are good indicators of meat shelf life [24–26] and many of them are products of fatty acid oxidation. Dietary fatty acids directly influence the fatty acid composition in the fish muscle [27–29], and therefore the inclusion of IMs and GM can potentially affect the volatile compound composition of the fish meat. Studies on the effect of IM and GM on the flavor volatile compounds in aquatic animals are limited because research on volatile compounds has been mainly focused on the effect of cooking methods, fresh/frozen conditions, and storage time on the meat. IM has been included in diets for chickens decreasing the levels of fatty acid peroxidation products, such as alcohols and aldehydes, suggesting less grassy, oily, fatty, and sweet odors in the meat [30]. To our knowledge, studies on the effect of IM inclusion on the volatile composition of aquatic animals have been limited to Atlantic salmon (*Salmo salar*), without significant changes [25]. GM has been included in feeds for beef and lamb, reducing the meat levels of volatile compounds that are linked to fast meat spoilage [15, 16]. GM has been included in feeds for rainbow trout [17], grass carp [31], and abalone [32], but the volatile compound profiles have been not elucidated in those studies.

The shell color of mollusck is determined by organic pigments, such as pyrroles, polyenes (carotenoids), melanin, and porphyrin [33] which mostly come from dietary sources. It is presumed that pigments are not synthesized de novo by mollusck [34], yet full understanding of shell pigmentation is still limited. In abalone species, the provision of diets supplemented with dietary pigments has shown to produce a change in the shell coloration, which can be manipulated according to market requirements. The dietary inclusion of the seaweed promoted a brownish colored shell in *Haliotis asinina* [35] *and Haliotis discus hannai* [36]. In addition, the supplementation of carotenoids (astaxanthin, canthaxanthin, and *β*-carotene) promoted shells with more yellow coloration in *Haliotis rufescens*.

This study aimed to assess the effects of the inclusion of IMfrom *T. molitor* and GM on the flavor volatile compound profile and shell color of the New Zealand abalone *Haliotis iris*. The key hypothesis of the study was that the fatty acid profile in *T. molitor* and GM will contribute to a significant change in the flavor volatile profile of the meat of *H. iris*. In addition, the supplementation of GM, which has anthocyanins as main pigment, will contribute to a change of coloration in the shell.

## 2. Materials and Methods

### 2.1. Animals, Experimental Setup, and Sample Collection

This study was conducted within a commercial abalone (*H. iris*) farm (The New Zealand Abalone Company) in Bluff, New Zealand. Healthy juveniles with initial mean weight and shell length of 1.1 ± 0.5 g and 21.5 ± 3.3 mm, respectively, were used in the feeding trial. The animals were 17-months old and were randomly selected from the farm stock. Fifteen plastic tanks containing 90 L of filtered seawater were stocked with 200 juveniles per tank. Abalone were randomly distributed among the tanks (three tanks per treatment) and were fed one of the experimental diets or commercial feed for 165 days. Abalone were fed in excess at 1.2%–2.2% of their body weight per day which was supplied in the late afternoon (1,600 hr). A flow-through water system was maintained the same during the feeding trial (water rate 1.5 L/min, water exchange of 40 times/day). The tanks were cleaned as per farming procedure every other day. Faeces, debris, and uneaten food residues were flushed out with the help of a water supply. Animals remained undisturbed in the tank while cleaning was performed. Water temperature and dissolved oxygen were measured using a dissolved oxygen meter (Handy Polaris TCP, Denmark) before and after cleaning abalone tanks. The dissolved oxygen meter was calibrated before use in “air-saturated” seawater according to manufacturer's instructions. During the feeding trial, the temperature ranged from 12.4 to 19.7°C, and dissolved oxygen was maintained between 86.3% and 104.1% oxygen saturation.

At the end of the feeding trial, 20 abalone were removed from each tank with the aid of a blunt knife and removed from their shells. Shells were cleaned with distilled water, air-dried, and collected for color measurement of the shells. For flavor volatile compound analysis, two animals were removed from each tank and then removed from their shells. The soft bodies of these animals were placed into 2 mL cryovial (Biostor™) and quenched in liquid nitrogen for 10 min and then stored in dry ice (−80°C) until further analysis.

### 2.2. Diet Preparation

Four experimental encapsulated diets and a commercial feed were used in the feeding trial. Experimental diets were developed using different levels of fish meal, IM, and GM were included as per Table 1. Diets were encapsulated and classified as: diet F (only containing fish meal as source of protein), FI (fish meal + IM), FG (fish meal + GM), and FIG (fish meal + IM + GM). A commercial feed (Marifeed S34) served as a control. Proximate composition of the experimental diets and commercial feed was determined according to AOAC [37]. This information is also reported in Table 2.

### 2.3. Volatile Compounds Analysis

The volatile compounds in abalone meat were extracted using the solid phase microextraction (SPME) and a gas chromatographymass spectrometry system. In brief, two animals from each experimental tank (six per treatment) were randomly selected for volatile compositional analysis at the end of the feeding trial. Since the size and wet weight of the animals were small, the whole body was used for analysis. Samples were kept at room temperature for 1 hr and chopped into small pieces. The chopped tissues from each individual abalone (0.50 g) were homogenized in 10 mL milliQ water using an UltraTurrax homogenizer (10,000 rpm, 1 min). The samples (2 mL) were placed in 10 mL flat bottom headspace vials fitted with a PTFE/silicone septum and screw cap (Supelco, Inc., Bellefonte, USA). A total of 0.7 g NaCl was added to increase the detection sensitivity of the solid-phase microextraction technique due to the “salting-out” effect. A volume of sodium azide solution (100 *µ*L) was added to each vial to obtain the final concentration of 0.02% w/v in each vial. A working solution of internal standard (1 ppm, 1,2-dichlorobenzene) was prepared using milliQ-water. A volume of 100 *µ*L of internal standard working solution was added to each vial. The vials were capped and transferred to gas chromatography–mass spectrometry equipment. The volatile compounds are extracted with a SPME fiber coated with 50/30 *μ*m layer of divinylbenzene–carboxen–polydimethylsiloxane (Supelco Co., Bellefonte, USA) that was inserted into the vial headspace at 50°C for 25 min. The SPME fiber was then removed from the headspace vial and injected into the GC–MS injector at 250°C for 20 min.

A gas chromatography system Agilent Technologies 7890B (Agilent Technologies, Santa Clara, CA, USA) attached to an Agilent Technologies 5977B MSD detector was used. The Agilent G3903-63008 GC column (Phase DB-FATWAX Ultra Inert) with the dimensions of 30 m × 250 *μ*m × 0.25 *μ*m was used. The sample analysis was randomized and automated using a Gerstel multipurpose sampler (MPS). The initial temperature of the column oven was held at 40°C for 2 min, then further increased to 240°C at a rate of 8°C /min and held for 3 min. The inlet temperature was held at 250°C with a total flow rate of 46.1 mL/min in splitless mode. The scanning range of mass spectra was 38–400 *m/z*, and electron ionization mode at 70 eV. Ion source temperature was 250°C, MSD transmission line temperature was 250°C and the quad set to 150°C. Volatile compounds were identified using the NIST 14 library using the MassHunter Acquisition software program. An alkane series C7–C30 (Supelco Co., Bellefonte, USA) was used to assist in compound identification.

### 2.4. Shell Color Determination

The color of the shell powder was characterized by direct readings on a color spectrophotometer (ColorFlex EZ, HunterLab, USA). Twenty abalone shells were washed after dissection with deionized water, air dried and ground using a grinder mill (Planetary Ball Mill PM100/Germany) for 3 min. Approximately 8 g of the shell powder was weighed for measurements and three replicates were used for each dietary treatment. The instrument was calibrated with green (*L*^*∗*^ = 51.67, *a*^*∗*^ = −26.62 and *b*^*∗*^ = 13.29) and white ceramic standard plates (*L*^*∗*^ = 93.94, *a*^*∗*^ = −0.95 and *b*^*∗*^ = 0.94). The color was reported in CIE system. *L*^*∗*^, *a*^*∗*^ and *b*^*∗*^ parameters indicate *L*^*∗*^ = lightness, *a*^*∗*^ = redness/greenness, and *b*^*∗*^ = yellowness/blueness, respectively.

## 3. Statistics

For shell color data, the normality was analyzed using the Shapiro–Wilk test. Data were statistically treated by one-way ANOVA test and Tukey post hoc test for multiple comparison when data followed a normal distribution. Homogeneity of variances was analyzed using Levene's test when samples followed a normal distribution. Nonparametric Kruskal–Wallis test was used when data did not follow a normal distribution followed by Dunn post hoc test for multiple comparison. The statistical package XLSTAT 2022.3.1 (Addinsoft, New York, USA) was used for those purposes. For volatile compounds, data were analyzed using the MetaboAnalyst 5.0 software. Before analysis through MetaboAnalyst, samples were normalized via auto scaling (mean centered and divided by the standard deviation of each variable) to make features more comparable. Comparison of feeding groups was made by one way analysis of variance (ANOVA) and principal component analysis (PCA) was used to identify groupings of all samples based on the underlying structure of the data. Tukey's post hoc tests were used to compare the mean differences between dietary treatments (*p* < 0.05).

## 4. Results

### 4.1. Volatile Compounds Analysis

**Figure 1:**
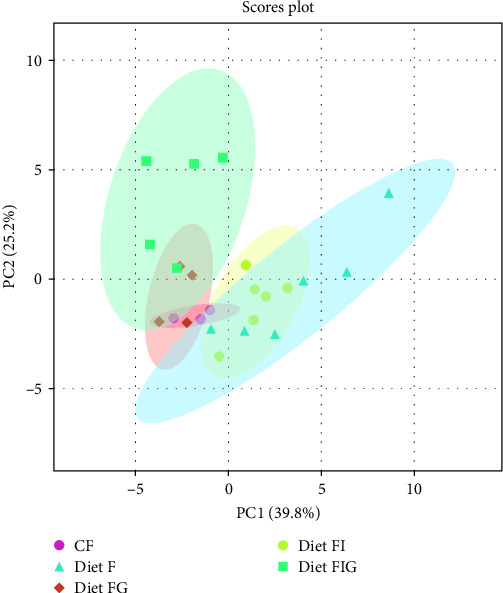
PCA plot samples based on their volatile compound profiles grouped by diets. Abbreviations: Diet F (fish meal based), FI (fish meal + insect meal), FG (fish meal + grape marc), FIG (fish meal + insect meal + grape marc), and commercial feed (CF).

The PCA results showed that the first component (PC1) and second component (PC2) explained 39.8% and 25.2% of the variance, respectively. The samples from different dietary treatments F and FIG were relatively separated according to their volatile profile in the PCA. Diet FIG and FG were not well separated, nor diets F and FI (Figure 1). Agreed with the results from Table 3, significant differences (*p* < 0.05) were seen in 18 volatile compounds among diets, and they contributed to the separation between diets with GM (FG and FIG) and without GM (F and FI). Significantly higher levels of 2,4-di-tert-butyl-phenol and cyclohexanone were observed in diet FIG compared to the rest of the diets. Diet F and FI had significantly higher levels of 1-octen-3-ol and 3-octanone compared to diet FG and FIG. Benzoic acid methyl ester was significantly higher in diet FIG compared to the other diets. Diet F had significantly higher values of pentanoic acid, 2-methyl anhydride; 2-pentanone; 2,3-pentanedione; 1-pentanol, and 2-penten-1-ol compared to diet FG and FIG. Diet FIG had significantly higher values of 2,2,4-trimethyl-1,3-pentanediol diisobutyrate. The compound 1-hexanol was significantly higher in diet FI compared to diet FG.

Diet FIG had significantly higher values of propanoic acid 2-methyl, 3-hydroxy-2,2,4-trimethylpentyl ester, and 3-heptanone compared to other experimental diets. Diet F and FI had significantly higher values of hexanal compared to commercial feed, but not to the other experimental diets. Diet FI had significantly higher values of 2-penten-1-ol compared to diet FG. Heptanal was significantly higher in diet F compared to CF, and 2-hexanone was significantly higher in diet FIG compared to CF.

**Figure 2:**
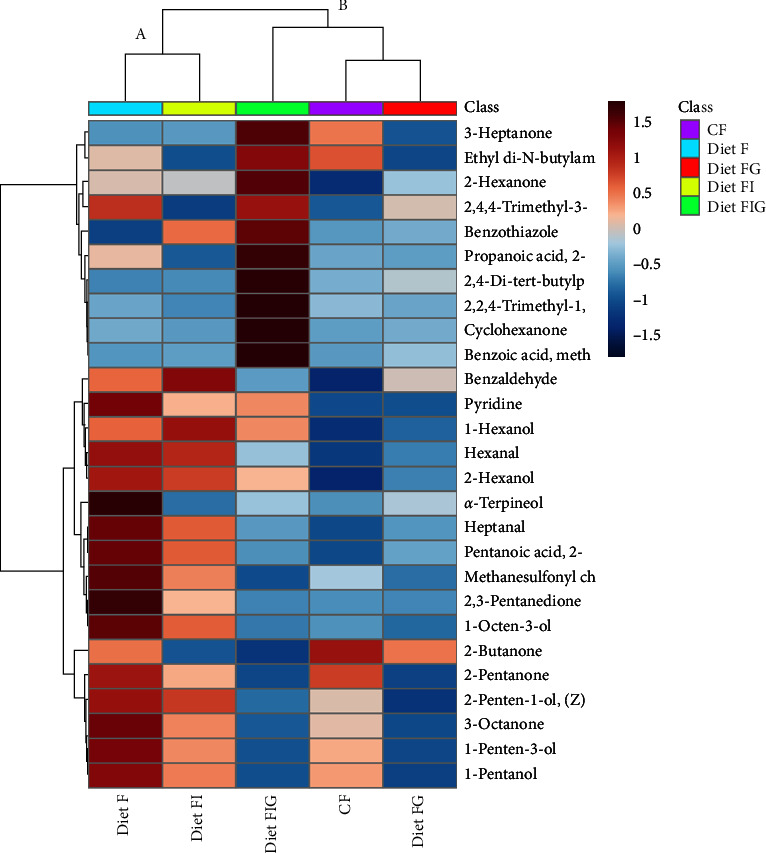
Heatmap of 27 metabolites found in abalone tissue. Only 18 compounds were found significantly different between the dietary treatments (one-way ANOVA, *p* < 0.05, FDR < 5%). Abbreviations: Diet F (fish meal based), FI (fish meal + insect meal), FG (fish meal + grape marc), FIG (fish meal + insect meal + grape marc), and commercial feed (CF). Metabolites abbreviations as per Table 3.

A heatmap of the volatile compounds showed that diet FIG had significant higher levels of ketones: 3-heptanone, 2-hexanone, and cyclohexanone compared to diet F which had high levels of ketones: 2,3-pentanodione, 2-pentanone, and 3-octanone (Figure 2). All aldehydes and alcohols were elevated in diet F compared to the other diets. Two clusters were differentiated: cluster A where samples fed diets F and FI were together, while cluster B showed samples fed diets FG, CF and FIG grouped together, which supported PCA ellipse separation. Cluster A was different from cluster B presenting elevated levels of alcohols, aldehydes, aromatic compounds, and ketones. Cluster B showed reduced levels of all compounds. Diet FIG, which was grouped under Cluster B, showed higher levels of ketones and esters compared to other diets in that cluster.

### 4.2. Shell Color

**Figure 3:**
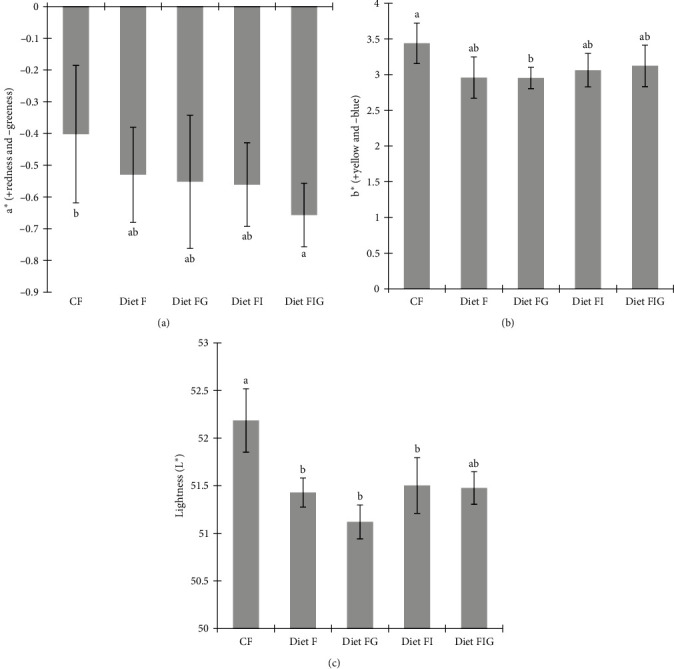
Shell colour in *Haliotis iris* receiving different dietary treatments in a 165-feeding trial according to (a) redness/greenness, (b) yellowness/blueness, and (c) lightness. Abbreviations: Diet F (fish meal based), FI (fish meal + insect meal), FG (fish meal + grape marc), FIG (fish meal + insect meal + grape marc), and commercial feed (CF). Data are presented as means ± SD of nine technical replicates. Different letters on top of the error bars denote significant differences resulting from *Tukey* post hoc tests (*p* < 0.05).

Results showed that redness and greenness were not significantly different among dietary treatments; however, a more negative value in diet FIG (−0.7 ± 0.1) indicated more redness compared to CF (−0.4 ± 0.2) (Figure 3(a)). There were no significant variations in terms of yellowness and blueness among the experimental diets. However, experimental diet FG (3.0 ± 0.2) showed significantly less yellowness and more blueness compared to CF (3.4 ± 0.3) (Figure 3(b)). There were no significant variations in shell lightness among the experimental diets with or without IM and GM (Figure 3(c)). The lightness (also called brightness) of the shells was significantly higher in animals feed commercial feed (CF) (52.2 ± 0.4) and FIG (51.5 ± 0.2) compared to other experimental diets F (51.4 ± 0.2), FG (51.5 ± 0.3), and FI (51.5 ± 0.3).

## 5. Discussion

*H. iris* is highly sought for the beauty of its shell color, which is considered a traditional art decoration in New Zealand. *H. iris* is mostly fed commercial diets in the farms, which promote faster growth, with fish meal being the main protein source. Fish meal has been considered an excellent protein source due to its palatability and the increased growth it promotes compared to seaweeds. The current market of aquafeeds is seeking fish meal replacements and alternative ingredients that can reduce the environmental impact, and simultaneously generate sustained economic profit by promoting the reutilisation of food wastes.

*H. iris* shells are highly valued for their use in jewelery and across traditional and modern indigenous Māori art. In this study, our results suggest that the inclusion of GM and IM did not significantly affect the lightness of the shells, nor the coloration of the shells. Although the lightness comparison between experimental diets and the commercial feed is not possible due to the lack of knowledge about the ingredients in the commercial feed, it was noted that abalone fed with the commercial diets (CF) had lighter shells compared to the experimental diets. The white coloration, which indicates more lightness, is associated with the calcium carbonate crystals that form aragonite and calcite structures in the shell of abalone [33]. Sometimes, commercial feed producers add compounds, such as phosphorous in the feed [42], to promote faster growth and calcium carbonate formation, especially during the early stages of formation [43], possibly increasing lightness, width, and height of the shell [44].

There were not significant variations among all experimental diets in terms of shell yellowness/blueness (*b*^*∗*^) and redness/greenness (*a*^*∗*^) coloration. Anthocyanins are water soluble pigments [45] and their colors can vary from red to blue depending on the pH value [46]. Studies have shown that anthocyanins from GM derivates modified the red and yellow color in fish muscle [47, 48]. However, the effect of dietary components on molluskan shells is not well understood [33, 49]. The colors of molluskan shells are determined by organic pigments, such as pyrroles, polyenes (carotenoids), melanin, and porphyrin [33] and their mechanism of affecting abalone has been observed but not elucidated [35, 50]. It is known that abalone do not produce pigments *de novo*, such as carotenoids, and instead these are obtained from the diet intensifying the yellow tones of *H. rufescens* shells [34]. The aquamarine blue pigment is mostly present in farmed *Haliotis cracherodiii* [51] and *H. iris* [52]. This pigment is relatively stable, unless in the presence of metallic ions, such as Fe^3+^ where the blue pigment become more yellow/green [51]. It is possible that the amount of GM included in the experimental diets would not have been enough to produce a significant red coloration in the shells of abalone. More studies on this matter including photographic image and pigment extraction are recommended.

For consumers, one of the most appealing features of a snail meat is the flavor, which is an indicator of consumer acceptance and preference [53]. Flavour is a complex phenomenon and starts with the visual perception and smell [54]. Once food is ingested, volatile compounds (odorants and irritants) from the food are transported to the odor receptors located in the nasopharynx [54]. Generally, the volatile compounds related to fish odor are generated by enzyme reaction, lipid oxidation, microbial action, and environmental reactions [55]. Examples of these volatile compounds are volatile aldehydes, alcohols, ketones, acids, amines, and aromatic compounds produced by oxidation of fatty acids.

Our results identified volatile compounds in abalone meat that are usually related to the “fishy” odor, such as hexanal; benzaldehyde, 1-hexanol, 1-octen-3-ol; 2,3-octanedione, 2-penten-1-ol; 1-penten-3-ol, “fatty” odor, such as heptanal and “earthy” odor, such as 1-pentanol [24, 39]. Those volatile compounds were found among all diets and only 18 were significantly different. Abalone fed diet F (fish meal as only source of protein) showed significantly higher levels of pentanoic acid, 2-methyl anhydride, 2-pentanone, 2,3-pentanedione, 1-pentanol, 2-penten-1-ol, 1-octen-3-ol, and 3-octanone compared to diets FG and FIG. This finding suggests that the presence of GM may have regulated the production of these lipid peroxidation products in abalone meat. GM has natural antioxidant and antimicrobial properties due to the high-tannin [56] and polyphenol content [57], mostly present in the skin and seeds of the grapes. Polyphenols and anthocyanins are free radical scavengers and prevent oxidation of PUFAs, such as linoleic acid [58], which in our study may have resulted in a significant reduction of the volatile compound 1-octen-3-ol. The inclusion of dietary GM to prevent the oxidation of animal meat is not novel. Turcu et al. [59] included GM at 3% and 6% in feeds for broilers which decreased the amount of thiobarbituric acid-reactive substances (TBARS) in the meat. Similarly, Ianni et al. [15] included GM at 10% in feeds for calves producing meat with reduced levels of hexanal, which is a considered a marker of lipid peroxidation [60, 61]. GM has shown to prevent microbial spoilage [62] and the formation of contaminants in meat, such as nitrosamines [63], acting as a natural food preservative [64].

The addition of IM in diet FI did not produce a significant difference in flavor volatile compounds compared to the diet F. Slight reductions of heptanal, 3-octanone, 1-penten-3-ol, and 1-pentanol in diet FI compared to diet F were observed, yet not significant. Typically, IM contains high levels of SFAs, and due to a slower oxidation compared to PUFAs, IMs have demonstrated to produce less peroxidation products compared to free-insect diets in the meat of chickens [30]. One possible explanation for the absence of significant variations between diet F and FI is the fatty acid profile present in *T. molitor* meal, which resembles fish meal and has been documented to be rich in PUFAs rather than SFAs [65]. A previous study in our research group indicated that the dietary inclusion of IM by 10% significantly increased the levels of *α*-linolenic acid, linoleaidic acid, arachidonic acid, and oleic acid compared to a fish meal-based diet in the meat of abalone. However, these changes may have not contributed to the increase of the lipid oxidation products in abalone meat. The lipid peroxidation products in animal meat have been mostly associated with higher contents of linoleic acid, which can cause the formation of more aldehydes [66]. In a previous study, the inclusion of IM (diet FI) have promoted significantly higher levels of precursors of linoleic acid (oleic acid and stearic acid) in the meat of abalone compared to a free IM/GM die. However, in this study the volatile compounds products were not significantly altered. The inclusion of IM produced significantly higher levels of alcohols 2-penten-1-ol, 1-octen-3-ol, and 1-hexanol compared to diet FG in abalone meat. Of them, 2-penten-1-ol has been found to be more associated with buttery, fish like, and green flavor, 1-octen-3-ol with mushroom, fermented, and potato flavor, and 1-hexanol with green and fish-like flavor [24]. From these compounds, 1-octen-3-ol is mostly attributed to crustaceans, due to the high content of fats in those animals. The presence of these compounds suggests that the inclusion of 10% IM influenced significantly the fishy-buttery characteristics of the meat compared to the diet with GM (FG). Further studies need to be performed using gradual increments of IM and GM and a taste panel.

Two volatile compounds, heptanal and hexanal, were found to be significantly different between the experimental diets and commercial feed. Heptanal was significantly lower in CF compared to diet F, and hexanal was significantly lower in CF compared to the diet F and diet FI. This finding indicates that abalone fed on diet F and FI (fish meal + IM) may have a more intense green, plant-like aroma usually related to meat freshness [67, 68] similar to when fishes are immediately harvested [69].

The present findings in this study indicated that the inclusion of IM from *T. molitor* did not produce significant changes in terms of flavor volatile compounds in abalone meat, whereas the inclusion of GM significantly reduced the volatile compounds related to lipid peroxidation. In addition, the inclusion of both, IM and GM did not have an impact on the shell's lightness, yellowness/redness/greenness, and blueness.

## 6. Conclusions

This is the first study to report differences in the volatile composition of the meat and shell color profile of *H. iris* after dietary treatment with diets that included IM from *T. molitor* and GM. The inclusion of IM did not significantly affect the volatile compound profile, whereas the inclusion of GM significantly reduced the production of most of the volatile compounds. Compounds such as 1-pentanol, 2-penten-1-ol, 2-pentanone, 2,3-pentanedione, and 1-hexanol were found to be significantly reduced with the inclusion of GM indicating a possible antioxidant effect which prevented lipid peroxidation in the raw meat of abalone. The dietary inclusion of IM and GM did not affect the lightness, redness/greenness, and yellowness/blueness of the ground shells. The insights gained from this study suggest that IM and GM are valuable ingredients for aquaculture feed in terms of cost-effective and sustainable production. However, further studies including both dietary ingredients are needed to evaluate meat flavor and shell coloration of adult market-size individuals.

## Figures and Tables

**Table 1:** Percentage (dry weight basis) composition of the experimental formulated diets (g/100 g).

	Diet			
Ingredients (g/100 g diet)	F	FI	FG	FIG
Fish meal^ƚ^	35	25	35	25
Insect meal^ǂ^	–	10	–	10
Corn meal	30	30	–	–
Grape marc^§^	–	–	30	30
Seaweed (dry) *Macrocystis pyrifera*^¶^	4	4	4	4
Starch (Native maize flour)^¥^	10	10	10	10

^ƚ^Fish meal supplied by Sandford, NZ. ^ǂ^Insect meal supplied by Mahurangi Technical Institute (MTI). ^§^Grape marc supplied by Bragato Research Institute, NZ. ^¶^Seaweed (*Macrocystis pyrifera*) supplied by Southern Clams. ^¥^Starch supplied by New Zealand starch. Abbreviations: Diet F (fish meal based), FI (fish meal + insect meal), FG (fish meal + grape marc), FIG (fish meal + insect meal + grape marc), and commercial feed (CF).

**Table 2:** Proximate composition of formulated diets and commercial feed.

	Diet				
Proximate composition	F	FI	FG	FIG	Commercial feed^¶^
Protein (%)	30.4 ± 0.1^ab^	27.3 ± 0.3^b^	30.8 ± 0.7^ab^	26.4 ± 2.0^b^	32.4 ± 2.7^a^
Carbohydrate (%)^ƚ^	47.9	50.8	45.4	49.3	46.34
Carbohydrate-reducing sugars (%)^ǂ^	28.9 ± 1.3^b^	32.0 ± 3.1^ab^	8.9 ± 1.9^c^	9.1 ± 1.1^c^	39.1 ± 4.7^a^
Total dietary fiber (%)	9.0 ± 0.2^b^	7.5 ± 0.1^c^	16.1 ± 0.1^a^	15.4 ± 0.3^a^	3.7 ± 0.3^d^
Lipid (%)	4.0 ± 0.2^c^	7.0 ± 0.6^a^	5.3 ± 0.5^b^	7.2 ± 0.3^a^	1.2 ± 0.3^d^
Ash (%)	13.5 ± 0.1^b^	11.9 ± 0.1^c^	14.5 ± 0.3^a^	12.9 ± 0.3^b^	6.8 ± 0.4^d^
Moisture (%)	4.2 ± 0.01^b^	3.0 ± 0.01^b^	3.9 ± 0.03^b^	4.2 ± 0.8^b^	10.7 ± 0.1^a^
Energy (J per g)^§^	18,100	20,400	18,900	20,200	15,900

Data represent means and standard deviation in dry matter of three technical replicates. ^ƚ^Carbohydrate proportion was calculated by difference 100—(moisture + protein + lipid + ash). ^ǂ^Carbohydrate was determined using reducing sugar method Anthrone. ^§^Total energy was calculated based on the physiological values at 5.6 kCal g^−1^ protein, 9.5 kCal g^−1^ lipid, and 4.1 kCal g^−1^ carbohydrates [38]. ^¶^Commercial feed used was Marifeed S34. Abbreviations: Diet F (fish meal based), FI (fish meal + insect meal), FG (fish meal + grape marc), FIG (fish meal + insect meal + grape marc), and commercial feed (CF).

**Table 3:** Significant different volatile compounds in abalone meat fed experimental diets and commercial feed using SPME-GCMS.

Compound	R.I^ƚ^	CAS #	Aroma description^ǂ^	Tukey's HSD
*Ketones*				
2-Hexanone	1,067.4	591-78-6	Fruity, buttery	Diet FIG > CF
2,3-Pentanedione	1,046.0	600-14-6	Butter, caramel, sweet	Diet F > CF; Diet FG < Diet F; Diet FIG < Diet F
3-Heptanone	1,140.1	106-35-4	Fruity, sweet	Diet FIG > Diet F; Diet FIG > Diet FG; Diet FIG > Diet FI
2-Pentanone	962.9	107-87-9	Fruity	Diet FIG < CF; Diet FG < Diet F; Diet FIG < Diet F
3-Octanone	1,241.8	106-68-3	Mushroom, earthy	Diet F > CF; Diet FG < Diet F; Diet FIG < Diet F; Diet FI > Diet FG; Diet FIG < Diet FI
2-Butanone	884.7	78-93-3	Fruity, green	n.s.
2,4,4-Trimethyl-3-(3-methylbutyl) cyclohex-2-enone	1,729.1	88,725-82-0	n.d.	n.s.
Cyclohexanone	1,278.8	108-94-1	n.d.	Diet FIG > CF; Diet FIG > Diet F; Diet FIG > Diet FG; Diet FIG > Diet FI
*Aldehydes*				
Hexanal	1,067.7	66-25-1	Fish-like, grassy	Diet F > CF; Diet FI > CF
Heptanal	1,172.1	111-71-7	Fish-like	Diet F > CF
Benzaldehyde	1,507.9	100-52-7	Almond, nutty	n.s.
*Alcohols*				
1-Octen-3-ol	1,441.8	3,391-86-4	Sweet, earthy	Diet F > CF; Diet FG < Diet F; Diet FIG < Diet F; Diet FI > Diet FG; Diet FIG < Diet FI
1-Penten-3-ol	1,160.6	616-25-1	Mushroom, fish-like	Diet FG < Diet F; Diet FIG < Diet F; Diet FI > Diet FG; Diet FIG < Diet FI
2-Penten-1-ol, (Z)	1,313.4	1,576-95-0	Mushroom, fish-like	Diet FG < Diet F; Diet FIG < Diet F; Diet FI > Diet FG
1-Pentanol	1,247.4	71-41-0	Earthy, mushroom	Diet FG < Diet F; Diet FIG < Diet F
1-Hexanol	1,347.2	111-27-3	Green, fish-like	Diet F > CF; Diet FI > CF; Diet FI > Diet FG
2-Hexanol	1,216.8	626-93-7	Fruity, fatty, winey	n.s.
*Ester*				
Benzoic acid, methyl ester	1,609.1	93-58-3	n.d.	Diet FIG > CF; Diet FIG > Diet F; Diet FIG > Diet FG; Diet FIG > Diet FI
Propanoic acid, 2-methyl-, 3-hydroxy-2,2,4-trimethylpentyl ester	1,858.7	77-68-9	n.d.	Diet FIG > Diet FG; Diet FIG > Diet FI
*Acid anhydride*				
2,2,4-Trimethyl-1,3-pentanediol diisobutyrate	1,874.6	6,846-50-0	n.d.	Diet FIG > CF; Diet FIG > Diet F; Diet FIG > Diet FG; Diet FIG > Diet FI
Pentanoic acid, 2-methyl-, anhydride	1,311.0	63,169-61-9	n.d.	Diet F > CF; Diet FI > CF; Diet FG < Diet F; Diet FIG < Diet F
Phenolic compounds				
*Aromatics*				
2,4-Di-tert-butylphenol	2,291.7	96-76-4	n.d.	Diet FIG > CF; Diet FIG > Diet F; Diet FIG > Diet FG; Diet FIG > Diet FI
Ethylbenzene	1,105.0	100-41-4		n.s.
Benzothiazole	1,941.7	95-16-9		n.s.
Pyridine	1,181.0	110-86-1		n.s.
*Terpenic derivatives*				
*α*-Terpineol	1,688.4	98-55-5		n.s.
Amine				
Ethyl di-N-butylamine	1,769.1	4,458-33-7		n.s.
*Sulphur compound*				
Methane sulfonyl chloride	1,193.4	124-63-0		n.s.

^ƚ^Volatile compound identified by SPME-GCMS analysis based on comparison with the RI and the mass spectra of standard compounds. ^ǂ^Reference aroma description based on Wang et al. [39], Jones et al. [24], Fukami et al. [40], Bai et al. [41], and http://www.thegoodscentscompany.com. n.d: No documented. n.s: No significant. Abbreviations: Diet F (fish meal based), FI (fish meal + insect meal), FG (fish meal + grape marc), FIG (fish meal + insect meal + grape marc), and commercial feed (CF).

## Data Availability

All the data in the article are available from the corresponding author upon reasonable request.
